# Need for more proactive use of pharmacists in the COVID-19 pandemic following lessons learnt from the Great East Japan Earthquake

**DOI:** 10.7189/jogh.10.020397

**Published:** 2020-12

**Authors:** Takanao Hashimoto, Toyoaki Sawano, Akihiko Ozaki, Masaharu Tsubokura, Takashi Tsuchiya

**Affiliations:** 1 Department of Pharmacy, Sendai City Medical Center, Sendai, Japan; 2 Department of Surgery, Sendai City Medical Center, Sendai, Japan; 3 Department of Radiation Health Management, Fukushima Medical University School of Medicine, Fukushima, Japan; 4 Department of Breast Surgery, Jyoban Hospital of Tokiwa Foundation, Iwaki, Fukushima, Japan

Maintaining an appropriate and sustainable supply of medical and health service products, including drugs, vaccines and sanitary materials, is of paramount importance in any crisis that impacts on public health in any population [1]. In the past, quackery spread during the Great Plague, and after World War 2 shortage of anti-infective agents and analgesics caused the spread of substandard and counterfeit drugs, leading to negative health impacts, including deaths [1]. In this regard, during the Coronavirus Disease 2019 (COVID-19) pandemic, which has spread rapidly around the world including Japan [2], constructing stable supply chains of resources such as drugs and other daily necessities should be essential for preventing undesired outcomes [1].

With any large-scale disaster, the shortage of medical and sanitary resources is a common problem [3,4]. As such, pharmacists may play a certain role in such circumstances, particularly with respect to the supply of drugs and other materials. The International Pharmaceutical Federation (FIP) has published a guideline (Responding to disaster: guidelines for pharmacy 2016), urging countries to develop rules in advance, based on national laws [5]. Nevertheless, pharmacists have not been ready to utilize their advanced skills fully in the current medical and social framework in Japan, especially in times of emergency, while physicians and nurses already have their specific roles in major disasters, and limited information is available on how pharmacists can be engaged in relief activities in large-scale disasters. In this context, we believe the experience of the 2011 Great East Japan Earthquake (GEJE) will provide helpful information to better define the role of pharmacists in ongoing COVID-19 pandemic and future disasters.

In the GEJE, which occurred on March 11, 2011, pharmacists, especially those in the affected areas, were engaged in disaster relief works [6]. For example, pharmacists and pharmaceutical wholesalers worked closely and played a vital role in maintaining the supply of drugs or sanitary materials in the GEJE [6]. Additionally, pharmacists, who took turns supporting the disaster-stricken areas and who had contributed to the construction of drug distribution systems following the GEJE, had been using handwritten notebooks to record their work because the regular functioning of medical facilities had been rendered inoperable due to the tsunami [6], These efforts might help guide efforts in helping to cope with the COVID-19 pandemic.

**Figure:**
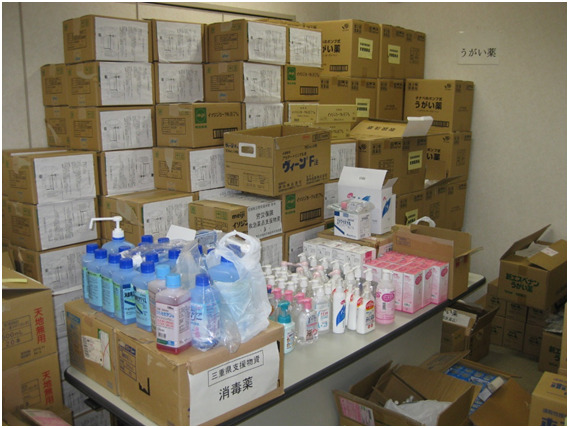
Photo: Piled-up relief materials after the Great East Japan Earthquake. Photo by the first author (T.H.), taken on April 20, 2011, location: Miyagi Prefectural Pharmacists Association Hall (Sendai City, Miyagi Prefecture). The photo shows piles of hand sanitizers from other areas. The amount of supportive and hygienic medicines and hygiene products was enormous and pharmacists on site were sorting them according to their needs.

Further, management of the living environment by pharmacists was also helpful during the GEJE, which could be applicable to the COVID-19 pandemic. Basically, pharmacists have controlled environmental factors in schools and other public facilities, such as temperature, humidity, water quality, dust level, and pests, in Japan [7]. For that reason, after the GEJE, pharmacists could have helped raise the awareness of evacuees to the appropriate use of disinfectants or sanitation of temporary toilets, as well as the temperature and humidity in evacuation sites. In Minamisanriku Town, Miyagi Prefecture, a municipality badly affected by the tsunami, evacuees were forced to live in narrow spaces because the main part of the evacuation center was used to store dead bodies and relief materials. Because water and sewage services were shut down for five months, the use of water other than drinking was limited to once a day. Hand sanitizer was very useful to disinfect and cleanse hands directly under the guidance of pharmacists in evacuation sites. It would be valuable during the COVID-19 pandemic for pharmacists to proactively intervene in the improvement of the living environment where infections are likely to occur.

Reflecting the GEJE, the training was started for “Pharmacy Disaster Life Support (PhDLS)” personnel who were destined to be responsible for the supply of drugs and the planning of pharmaceutical support activities in a large-scale disaster [8]. Large quantities of relief materials were haphazardly piled up at evacuation centers after the GEJE and on-site health care professionals (HCPs) were confused due to the mix of brand name drugs and generics. Pharmacists had checked the ingredients of drugs and suggested alternatives to prescriptions written by physicians based on availability. The practical role of such pharmacists is one of the most insightful and important points for future disasters preparedness.

However, it appeared that the lessons learnt in past major disasters, particularly with regard to drug or sanitary material were not applied during the COVID-19 outbreak in Japan. The role of pharmacists in the COVID-19 pandemic, including drug supply and pharmacotherapy planning (**Table 1**), has been gradually reported [9]. On the other hand, according to Japanese guideline regarding “temporary pharmaceutical distribution at the large-scale disaster” [6], under the leadership of local governments, physicians, pharmacists, local pharmaceutical wholesalers and others have to work together and build systems to ensure drug delivery, with no shortages or coverage at the time of a disaster. However, supply systems had not functioned effectively during the COVID-19 outbreak, and from March to June 2020, some HCPs were forced to use the same face masks for several days and wear rain gear or other alternatives to proper Personal Protection Equipment (PPE) and as a result, operations for infection control were inadequate in some hospitals [2]. Additionally, during the COVID-19 outbreak, PhDLS [8] had not been engaged in any activities, as far as we know. Those problems must be reviewed and we HCPs and stakeholders should revisit effective measures to prevent from such inadequate responses in future.

**Table 1:** The role of pharmacists in the supply of drugs and sanitary materials and management of the living environment during the COVID-19 pandemic based on GEJE’s experience

	Problems during the COVID-19 pandemic	Countermeasures based on the experience of the GEJE
Supply of drugs and sanitary materials	● Disruption of medical supply system	● Temporary pharmaceutical distribution centers tailored to HCPs’ needs.
	● HCPs were forced to use the same face masks for several days	● Building supply systems in cooperation with local pharmaceutical wholesalers and other organizations.
	● Improper use of Personal Protection Equipment (PPE)	● Training of Pharmacy Disaster Life Support.
Management of the living environment	● Insufficient knowledge about how the infection develops among citizens	● Construction of hygiene management at evacuation centers
	● Scarce preparedness toward other disaster in COVID-19 era	● Raising the awareness of evacuees to the appropriate use of disinfectants or sanitation of temporary toilets
		● Promotion of hand sanitizer in narrow evacuation sites

GEJE – Great East Japan Earthquake, HCP – health care professional

The current COVID-19 pandemic is projected to last for several years, based on past experiences with the Spanish Flu [10], and will require additional efforts of HCPs. In the aftermath of the GEJE, pharmacists contributed to the supply of drugs and improvement of the environment to meet the needs of patients in the affected areas [6]. The participation of pharmacists could help support the treatment for patients in the COVID-19 pandemic.

Finally, with regard to the supply of drugs and sanitary materials and management of the living environment, the table summarizes the problems encountered during the COVID-19 pandemic and the measures taken based on GEJE’s experience [6]. We hope that lessons learned from the GEJE will be one of the catalysts for rethinking the role of pharmacists during the COVID-19 pandemic.
